# Dacomitinib, but not lapatinib, suppressed progression in castration-resistant prostate cancer models by preventing HER2 increase

**DOI:** 10.1038/s41416-019-0496-4

**Published:** 2019-06-18

**Authors:** Maitreyee K. Jathal, Thomas M. Steele, Salma Siddiqui, Benjamin A. Mooso, Leandro S. D’Abronzo, Christiana M. Drake, Young E. Whang, Paramita M. Ghosh

**Affiliations:** 1 Department of Urology, University of California Davis, Sacramento, CA United States; 2 VA Northern California Health Care System, Mather, CA United States; 3 Department of Biochemistry-Molecular Medicine, University of California Davis, Sacramento, CA United States; 4 Department of Statistics, University of California Davis, Davis, CA United States; 5 Lineberger Comprehensive Cancer Center, University of North Carolina, Chapel Hill, NC United States

**Keywords:** Prostate cancer, Prostate cancer

## Abstract

**Background:**

Despite overexpression of the ErbB (EGFR/HER2/ErbB3/ErbB4) family in castration-resistant prostate cancer (CRPC), some inhibitors of this family, including the dual EGFR/HER2 inhibitor lapatinib, failed in Phase II clinical trials. Hence, we investigated mechanisms of lapatinib resistance to determine whether alternate ErbB inhibitors can succeed.

**Methods:**

The CWR22 human tumour xenograft and its CRPC subline 22Rv1 and sera from lapatinib-treated CRPC patients from a previously reported Phase II trial were used to study lapatinib resistance. Mechanistic studies were conducted in LNCaP, C4-2 and 22Rv1 cell lines.

**Results:**

Lapatinib increased intratumoral HER2 protein, which encouraged resistance to this treatment in mouse models. Sera from CRPC patients following lapatinib treatment demonstrated increased HER2 levels. Investigation of the mechanism of lapatinib-induced HER2 increase revealed that lapatinib promotes HER2 protein stability, leading to membrane localisation, EGFR/HER2 heterodimerisation and signalling, elevating cell viability. Knockdown of HER2 and ErbB3, but not EGFR, sensitised CRPC cells to lapatinib. At equimolar concentrations, the recently FDA-approved pan-ErbB inhibitor dacomitinib decreased HER2 protein stability, prevented ErbB membrane localisation (despite continued membrane integrity) and EGFR/HER2 heterodimerisation, thereby decreasing downstream signalling and increasing apoptosis.

**Conclusions:**

Targeting the EGFR axis using the irreversible pan-ErbB inhibitor dacomitinib is a viable therapeutic option for CRPC.

## Background

Recurrent or metastatic prostate cancer (PCa) is treated with androgen deprivation therapy (ADT) but patients inevitably relapse, indicating onset of castration-resistant PCa (CRPC). Subsequent FDA-approved treatment options include chemotherapy, immunotherapy and androgen receptor (AR) signalling inhibitors but patients eventually fail these agents. The continued efficacy of the AR inhibitors in CRPC illustrates the central role played by the AR in PCa growth and survival.^1–3^ Abiraterone, an androgen synthesis inhibitor, and enzalutamide, a potent AR antagonist, extend progression-free and overall survival of metastatic CRPC patients in both the post- and pre-chemotherapy settings.^4–7^ However, due to inevitable development of resistance to these agents, CRPC remains incurable and novel therapies are needed.

We previously showed that upregulation of the receptor tyrosine kinase (RTK)s of the epidermal growth factor receptor (EGFR) family was a major cause of PCa recurrence following AR inhibition.^8^ The EGFR family is comprised of four members: EGFR/ErbB1, HER2/ErbB2, HER3/ErbB3 and HER4/ErbB4 that are activated by ligand-binding (except HER2), followed by dimerisation and phosphorylation.^9^ HER2 exists in a constitutively ‘open’ conformation and is the preferred dimerisation partner for EGFR and HER2. ErbB3 itself has weak, intrinsic kinase activity but acts as a supporting kinase for EGFR and HER2.^10^

Unlike many other cancers, PCa tumours express high EGFR and ErbB3, low HER2, and mostly lack ErbB4 expression.^11,12^ Single inhibitors of EGFR and HER2 (e.g. gefitinib, erlotinib and trastuzumab) that were successful in other cancers, failed in PCa clinical trials^13–17^ and our data implicated ErbB3 signalling in this resistance.^18^ Lapatinib (the first, FDA-approved, small-molecular dual-HER2/EGFR tyrosine kinase inhibitor (TKI) for treatment of HER2+/ErbB2+ breast cancers^19^) was ineffective in PCa clinical trials. Single-agent lapatinib, though well-tolerated, showed no overall positive effect in CRPC^20^ or in hormone-sensitive PCa (HSPC).^21,22^ The purpose of the current study was to understand mechanisms of lapatinib resistance in CRPC in order to better design alternative techniques that would benefit CRPC patients.

In this paper, we use an animal model of CRPC, which realistically replicated the lack of efficacy of lapatinib in human patients. Using this model, we demonstrated an increase in HER2 upon lapatinib treatment that correlated with resistance to this therapy. Similar to the observations in this model, patients with CRPC who were treated with lapatinib during a Phase II single arm clinical trial^20^ exhibited significantly increased serum HER2 that correlated with PSA increase. An unrelated in vitro model also showed similar increase in HER2 with lapatinib treatment and was non-responsive to this treatment; however, responsiveness was restored upon downregulation of HER2 implicating HER2 upregulation in the non-response to this treatment. This increase in HER2 is caused by increased protein synthesis, not increased transcription, as well as decreased proteasomal degradation following lapatinib treatment. Investigation of the mechanism by which HER2 upregulation induced resistance to lapatinib showed that under conditions of androgen deprivation, lapatinib upregulated EGFR/HER2 dimerisation, which enhanced downstream signalling via ERK phosphorylation. Unlike lapatinib, the pan-ErbB inhibitor dacomitinib which has recently been FDA approved for non-small cell lung cancer,^23,24^ prevented HER2 protein synthesis, membrane localisation and eventual EGFR/HER2 heterodimerisation without compromising membrane integrity. Dacomitinib, unlike lapatinib, suppressed CRPC cell growth, downregulated EGFR/HER2 heterodimers and induced apoptosis. Taken together, these results indicate that dacomitinib may be effective in CRPC despite the failure of lapatinib.

## Methods

### Animal studies

Four to 5-week-old Balb/c athymic nude-Foxn1nu (nu/nu) male mice were obtained from Harlan Sprague Dawley, Inc. Suspensions of CWR22 cells or CWR22-Rv1 cells were mixed in 50% Matrigel-solubilised basement membrane (BD Biosciences) and xenografts established by s.c. injections of 2.5 million cells/site into the flank. When palpable tumours were observed, animals were gavaged 5 days a week with vehicle (50:50 v/v solution of PBS and 0.5% Tween-20) or 50 mg/kg lapatinib. Three days after start of drug regimen, the animals were castrated by bilateral scrotal excision, following isoflurane-anesthetisation. Control animals were sham-operated by opening the animals surgically, but no tissues were removed. Drug administration was continued post-surgery and mice were sacrificed when tumour size exceeded 150 mm^3^ in any one dimension or at the end of the study period. Mouse weight and tumour size was recorded daily. Tumours were collected and partly formalin fixed and paraffin-embedded (FFPE) for immunohistochemistry while the remaining tumours were snap-frozen in liquid nitrogen, then lysed for immunoblotting.

### Patient specimens, serum EGFR and HER2 measurements and data analysis

Patient sera were collected from a previously reported, prospective, multi-institutional, open-label, single arm phase II study of lapatinib.^20^ Briefly, CRPC patients who had not received chemotherapy were included in the study. The primary objective was to determine the proportion of patients demonstrating >50% decline in PSA after treatment with lapatinib at a dose of 1500 mg once daily. The primary end point was a >50% confirmed PSA decline from baseline. Serum samples obtained before and after starting lapatinib were available in a subset of patients (14 out of 29 total) and were stored at −80 C. Samples were analysed for levels of extracellular domains of EGFR and HER2 using an enzyme-linked immunosorbent assay (ELISA) by a commercial laboratory (Pathway Diagnostics, Dorking, England). Characteristics of patients whose sera was used in the study are shown in Table S1.

### Cell culture and materials

Human prostatic carcinoma epithelial cell lines LNCaP, 22Rv1 (ATCC, Manassas, VA) and C4-2 (MD Anderson, Houston, TX), were cultured in RPMI 1640 medium with 10% fetal bovine serum (FBS) (Gemini Bioproducts, West Sacramento, CA) and 1% antibiotic-antimycotic solutions (Gibco/Thermo Fisher, Waltham, MA). Castration was simulated with culture of the cells in charcoal stripped serum (CSS) where androgen levels were significantly suppressed, compared to those cultured in fetal bovine serum (FBS), which was replete with androgens.^25^ CWR22 tumours were generously donated by Dr Clifford G. Tepper, Department of Biochemistry and Molecular Medicine, University of California, Davis. Molecular characteristics of these prostate cancer model systems are shown in Table S2. Recombinant human epidermal growth factor (EGF) and recombinant human heregulin-1β (HRG) were from PeproTech (Rocky Hill, NJ); Lapatinib and dacomitinib from LC Laboratories (Woburn, MA); Cycloheximide and staurosporine from Cell Signaling Technologies (Beverly, MA); Propidium iodide and annexin V APC from Molecular Probes/Invitrogen (Carlsbad, CA). The rabbit monoclonal antibodies anti-EGFR, HER2, ErbB3, and rabbit polyclonal androgen receptor, Lamin A/C, E-cadherin, Akt, phospho-Akt (Ser473), ERK1/2, phospho-ERK1/2 (Thr202/Tyr204) and α-tubulin were from Cell Signaling Technology (Beverly, MA). Mouse monoclonal antibodies (used for immunoprecipitation reactions) to EGFR (sc-373746), HER2 (sc-33684) and ErbB3 (sc-7390) were from Santa Cruz (Dallas, TX).

### Plasmids, primers and siRNA

EGFR, HER2/ErbB2 and HER3/ErbB3 primer sequences are listed in Table S3. Cells were transiently transfected using Lipofectamine 2000 reagent (Invitrogen, Grand Island, NY) according to the manufacturer’s instructions. SiRNAs (Table S4) to EGFR (M-003114-03-0010), HER2 (M-003126-04-0005), ErbB3 (M-003127-03-0005) and non-specific control sequences (D-001206-13-20) were obtained from Dharmacon (siGENOME SMARTPool) (Lafayette, CO).

### Statistical analyses

Difference in levels of protein expression among patients were calculated by Analysis of Variance (ANOVA). Waterfall plots were created in Excel and represent percent change in EGFR or HER2 levels post-treatment with lapatinib compared to pre-treatment levels. Patients were classified according to change in PSA after one cycle of treatment—those with >2-fold increase were classified as “high” PSA while those with ≤2-fold increase in PSA were classified as “low” PSA group. EGFR and HER2 changes in the “low” and “high” PSA groups were compared by Wilcoxon matched-pairs signed rank test. Correlations between PSA and markers were estimated using Spearman correlation coefficient. Tumour growth of CWR22 and 22Rv1 xenografts was compared by calculating the slope of the curve for each condition using the GLM procedure in SAS system. Marker levels were compared between groups using Kruskal–Wallis tests to test globally for any differences among groups, followed by pairwise comparisons using Wilcoxon rank sum tests in the case of a significant Kruskal–Wallis test. Neither EGFR nor HER2 was expressed in the nucleus in the mouse study, therefore, analysis was carried out only for cytoplasmic EGFR and HER2. A simple tabulation revealed that the majority of observations were concentrated at a few values only. Therefore, EGFR and HER2 were recoded into high (values great then 2) or low (values between zero and 1.5). Statistical analysis was carried out comparing expression of EGFR and HER2 for intact vs castrated mice by line and by receipt of drug. Two types of models were used. Logistic regression using the CATMOD procedure in SAS was carried out separately by line with the outcome expression of EGFR, respectively, HER2 comparing mice on drug vs no drug and castrated vs intact mice. The association of EGFR and HER2 with receipt of drug and castration status was also analysed using analysis of variance. While both variables have only a limited number of values, residual analysis did not show strong departures from model assumptions. For cell line studies, MTT assays were conducted on three biological replicates and significant differences compared using Welch’s ANOVA in Graphpad Prism 7. Growth curves were created in Excel.

### Other methods

Western blotting, qPCR, MTT viability assays, immunohistochemistry, immunofluorescence and flow cytometry are described in Supplementary Materials.

## Results

Figures S1–S7 are to be found in Supplementary Material.

### Lapatinib increases protein levels of HER2 in hormone-sensitive and castration-resistant human PCa xenografts

**Fig. 1:**
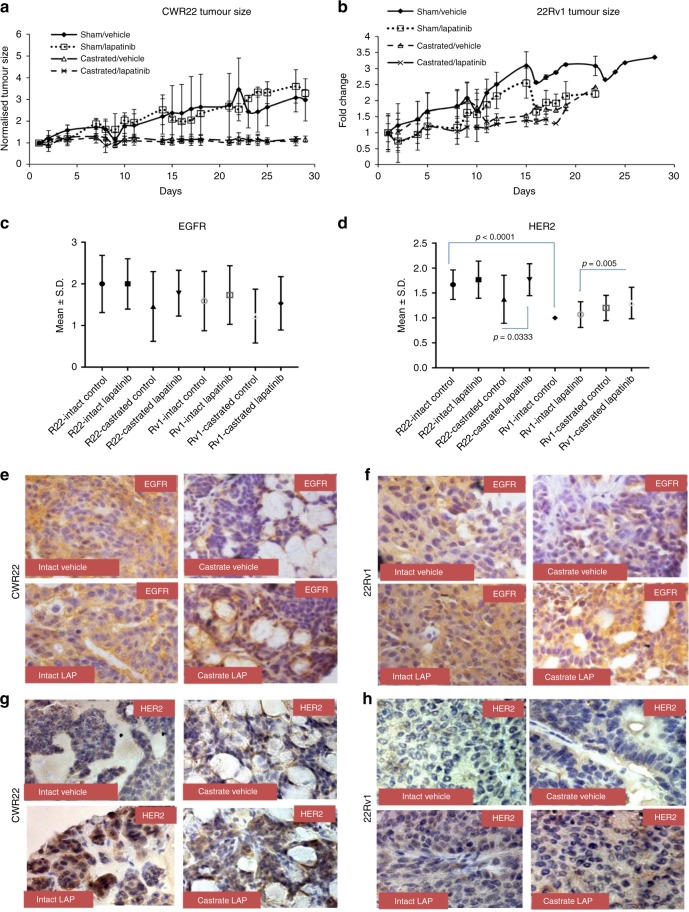
Lapatinib causes an increase in HER2 levels in xenografts established from human-derived tumour lines. **a** Four to 5-month-old athymic nu/nu mice (*n* = 6/group) were subcutaneously inoculated with CWR22 tumour tissue, after 3 days, the animals were castrated or underwent a sham operation (intact) and further treated with 50 mg/kg lapatinib daily or a placebo. The animals were followed for 26 days following surgery and euthanised (i) at the end of the experiment or (ii) if the tumours exceeded 200 cm^3^ or (iii) the animals were suffering. While castration severely limited the size of the tumours (*p* = 0.0012), the effect of lapatinib was minimal. Each point represents the median fold change in tumour size over the size the day of castration. Error bars represent standard deviation. **b** The CWR22Rv1 tumour line, derived from a relapsed CWR22 tumour in a castrated mouse, is resistant to castration and showed no effect of lapatinib either. Note that in this group, all animals were euthanised on day 19 post-castration, except one control mouse who continued on to day 26 to compare with the CWR22 group. **c, d** Numerical representation of baseline in vivo analyses of intratumoral **c** EGFR and **d** HER2 protein levels in human prostate cancer xenografts. Each point represents mean ± s.d. of six mice treated with the conditions indicated. **e–h** Immunohistochemical analyses of subcutaneously-injected **e, g** CWR22 or **f, h** CWR22-Rv1 tumours treated as indicated and stained with **e, f** EGFR or **g, h** HER2. Pictures are magnified at ×40 and are representative examples of each group. Brown staining indicates positivity

Immunodeficient male mice were subcutaneously implanted with the well-characterised CWR22 (hormone-sensitive) tumour line^8,18,26,27^ and its relapsed, CRPC subline 22Rv1.^28^ Half the animals were left intact and half were subjected to castration. Castration reduced tumour volume in CWR22 (mean tumour growth rate 3.88 ± 2.23 in intact vs −0.02 ± 0.55 in castrated mice, *p* < 0.0001) (Fig. 1a) but not 22Rv1 tumours (mean tumour growth rate 7.55 ± 3.47 in intact vs 10.62 ± 8.59 in castrated mice, *p* = 0.3465) (Fig. 1b). Lapatinib failed to reduce tumour size in either model (CWR22 or 22Rv1) either in intact or in castrated mice (*p* > 0.05) (Fig. 1a, b). Lapatinib was well-tolerated by all mice, with constant body weights recorded throughout the treatment period (Fig. S1A). Overall the rate of tumour growth in the mice bearing CWR22 tumours (mean growth rate = 2.24 ± 3) was significantly lower than that in mice bearing 22Rv1 tumours (mean growth rate = 7.58 ± 4.99) (*p* = 0.0001) **(**Fig. S1B**)**. Analyses of the expression of the EGFR family by immunohistochemistry revealed that expression of EGFR showed association neither with lapatinib treatment nor with castration status in either line (Fig. 1c). Nevertheless, expression was slightly higher in animals on lapatinib and in intact animals in both lines. Expression of HER2 was low for all animals in 22Rv1 tumours compared to CWR22 (*p* < 0.0001) (Fig. 1d) (illustrated in Fig. 1e–h). Examination of corresponding tumour lysates supported these observations (Fig. S2A). In placebo fed mice, castration did not significantly affect EGFR or HER2 levels in the tumour (Fig. S2B); however, among those bearing 22Rv1 tumours and treated with lapatinib, HER2, but not EGFR levels were elevated in castrated mice, compared to intact mice (*p* = 0.005). HER2 expression was higher in mice who received lapatinib in CWR22 tumours (*p* = 0.0333) (Fig. 1d), but no corresponding change in EGFR was observed. There was a more pronounced effect in 22Rv1 tumours where expression was higher in castrated mice (*p*-value = 0.0074). In support of these results, immunoblotting of tumour lysates demonstrated comparable EGFR and increased HER2 in castrated CWR22 tumours treated with lapatinib (Fig. S2D), but no change in intact mice (Fig. S2C). The tumours did not express ErbB4 protein (not shown).

### Patients with castration-resistant PCa treated with lapatinib demonstrate an increase in serum HER2 levels

**Fig. 2:**
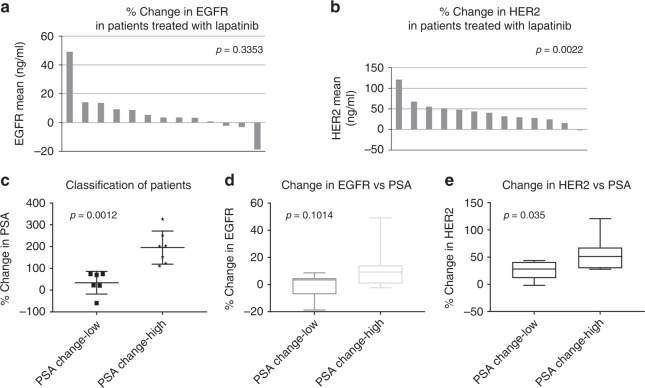
Lapatinib treatment induces higher serum levels of HER2 in patients with castration-resistant prostate cancer. **a** Waterfall plot demonstrating the change in EGFR levels in the serum of patients enrolled in a previously reported single-arm open-label Phase II trial on lapatinib (*n* = 13).^20^ Note that only 3/13(23%) experienced a decrease in serum levels of EGFR. Median % change in EGFR (pre- vs post-treatment) was +3.48% (range: −18.7% to +49.1%), mean increase: 6.66%; however, this increase was not significant (*p* = 0.3353). **b** In contrast, only 1/13 = 7.7% experienced any decrease in HER2. Median % change in HER2 (pre- vs post-treatment) was +40.35% (range: −2.02% to + 120.83%); mean increase: 42.6%, *p* = 0.0022. **c** Patients were classified according to “low” PSA changes (PSA change less than 2-fold) that of pre-treatment levels following lapatinib treatment at the time of progression, vs “high” PSA changes—(PSA change > 2-fold), providing two non-overlapping groups (*p* = 0.0012)**. d** Percent changes in EGFR were not significantly different between the “low” and “high” PSA groups (*p* = 0.1014) **e** but percent change in HER2 correlated strongly with change in PSA for the duration of the treatment (*p* = 0.035)

To identify a cause for lapatinib failure in a Phase II trial in CRPC patients,^20^ we investigated whether lapatinib affected serum levels of targets EGFR and HER2. Serum was available from 14 of 29 patients who participated in this trial; their characteristics are described in Table S1. Evaluation of serum EGFR and HER2 in 13 patients whose serum was available both pre- and post-treatment revealed that 10 of 13 (76.9%) experienced increased EGFR following lapatinib treatment (mean increase pre-treatment to post-treatment: 6.66 ± 15.29%), but the variation was large enough that the difference was not significant (*p* = 0.3353) **(**Fig. 2a**)**. On the other hand, all but one patient demonstrated a significant increase in serum HER2 levels (12 of 13 patients; 92.3%; mean increase pre-treatment to post-treatment: 42.6 ± 29.64%, *p* = 0.0022) (Fig. 2b). Overall, the majority of patients experienced increases in EGFR and HER2 (72%); three showed increased HER2 without increased EGFR and only one showed increased EGFR without increased HER2 (Fig. S3). Note that the mean baseline level of HER2 (8.71 ± 1.41 ng/ml) in the pre-treatment sera was substantially lower than the corresponding levels of EGFR (44.68 ± 6.01 ng/ml). Higher baseline levels of EGFR were observed in all patients compared to HER2 (*p* = 0.0002). Patients were classified according to “low” PSA changes, where post-treatment PSA levels were less than double (<100%) that of pre-treatment levels following lapatinib treatment, vs “high” PSA changes—where the post-treatment PSA levels more than doubled (≥100%) following lapatinib treatment compared to pre-treatment values (Fig. 2c). Change in post- vs pre-treatment EGFR was not significantly different between the “low” and “high” PSA groups (*p* = 0.1014) (Fig. 2d) but corresponding change in HER2 (Fig. 2e), was strongly related to change in PSA for the duration of the treatment (*p* = 0.035). Neither EGFR nor HER2 correlated with absolute PSA levels (not shown).

### Increase in HER2 levels in multiple human PCa cell lines enhances EGFR/HER2 heterodimerisation and promotes resistance to lapatinib

**Fig. 3:**
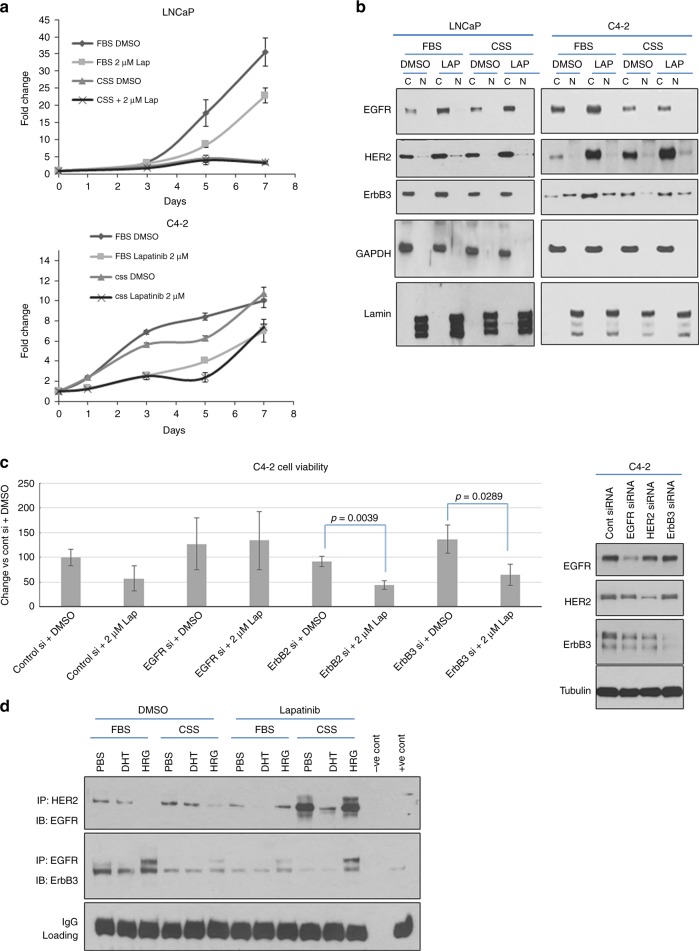
Lapatinib-induced increases in HER2 levels in multiple human prostate cancer cell lines correlates with EGFR/HER2 heterodimerisation and resistance to treatment. **a** (top) Hormone-sensitive (HS) LNCaP prostate cancer cells were treated with vehicle (DMSO) or lapatinib (Lap) and either deprived of androgens/growth factors in culture medium (CSS, with very low levels of steroid hormones and growth factors) or not (FBS, contains sufficient levels of steroid and growth hormones). By MTT assay, LNCaP cells exhibit a decrease in viability when cultured in CSS, compared to culture in FBS; however, they were not affected significantly by the presence of 2 µM lapatinib. (bottom) In contrast, C4-2 cells, a CRPC subline derived from LNCaP tumours growing in castrated nude mice, did not respond to removal of growth factors and steroid hormones by culture in CSS, or by 2 µM lapatinib. C4-2 cells therefore represent a group of cells that developed acquired resistance to lapatinib. Each point represents mean ± S.D. of three biological replicates. **b** Western blots demonstrating effects of DMSO or 2 µM lapatinib in LNCaP and C4-2 cells cultured in FBS or CSS. All treatments occurred for 72 h. Cells were separated into nuclear (N) and cytoplasmic (C) fractions. Lapatinib increased cytoplasmic EGFR and HER2 in low passage LNCaP (HS) and C4-2 (CRPC) cell lines. **c (**left) MTT assay demonstrating that silencing EGFR, HER2 or ErbB3 with sequence-specific siRNA decreased cell viability and sensitised C4-2 cells to lapatinib. Note that C4-2 cells are more sensitive to the inhibitory effect of decreasing HER2 (*p* = 0.0016) and ErbB3 (*p* = 0.0029) levels compared to decreasing EGFR levels (*p* = 0.0209). **c (**right) Western blot panel indicating specificity and effectiveness of RTK knockdown. **d** Co-IP demonstrating effect of lapatinib on heterodimerisation of the EGFR family of RTKs. Cells were cultured in FBS or CSS and treated for 72 h with DMSO or 2 µM lapatinib. At the end of that period, 1 nM DHT or 50 ng/ml HRG was added for 30 min. Cell lysates were immunoprecipitated (IP), the immunoprecipitates run on SDS-PAGE and immunoblotted (IB) as indicated. “−ve cont”: Negative control—no antibody was added. “+ve cont”: positive control—lysate only

We next investigated whether increase HER2 promoted lapatinib resistance in CRPC. For this purpose, we utilised the hormone-sensitive PCa (HSPC) LNCaP cells and its castration-derived subline C4-2 (CRPC) cell line. In LNCaP cells (Fig. 3a top) but not in C4-2 (Fig. 3a bottom), cell viability was decreased by culture in low-androgen media (CSS) compared to optimum androgen levels (FBS), similar to comparable observations made in vivo. Neither cell line was significantly affected by treatment with lapatinib, therefore phenocopying the effects seen in the in vivo models. Seventy-two hour treatment with 2 µM lapatinib significantly increased HER2, but not EGFR protein levels, in C4-2 cells both in FBS and CSS (Fig. 3b). In contrast to CWR22 tumours, LNCaP cells experienced increase in EGFR upon lapatinib treatment, likely reflecting variability of response of various HSPC models; whereas the response of CRPC models to lapatinib showing HER2 but not EGFR increase was consistent in all models used.

Silencing HER2 and ErbB3, but not EGFR, restored the ability of lapatinib to suppress cell viability in C4-2 cells, thereby confirming a significant role for these RTKs in lapatinib resistance (Fig. 3c, left). Knockdown of the RTKs were verified by western blotting (Fig. 3c, right). Since both HER2 and ErbB3 affected lapatinib sensitivity, we investigated these RTKs further. To examine the role of ErbB3, cells were stimulated with dihydrotestosterone (DHT)—a strong AR ligand, as control, or with heregulin-1β (HRG), a ligand for ErbB3. DHT alone did not alter EGFR dimerisation with either HER2 or ErbB3, whereas HRG stimulated EGFR/ErbB3 dimerisation. Similar to lapatinib-stimulation of HER2 levels only in castrated mice bearing 22Rv1 tumours (Fig. 1d), we saw increased EGFR/HER2 (but not EGFR/ErbB3) heterodimerisation with lapatinib only in CRPC cells in CSS (Fig. 3d). EGFR/HER2 heterodimerisation increased by CSS was reversed by DHT, but not HRG, indicating a role for AR, but not ErbB3, in this increase (Fig. 3d). Taken together, these data indicate that resistance to lapatinib results primarily from an increase in HER2, which increased EGFR/HER2 heterodimerisation in an androgen-dependent manner.

### The pan-ErbB inhibitor Dacomitinib, unlike the dual EGFR/HER2 inhibitor lapatinib, does not increase EGFR or HER2 levels

**Fig. 4:**
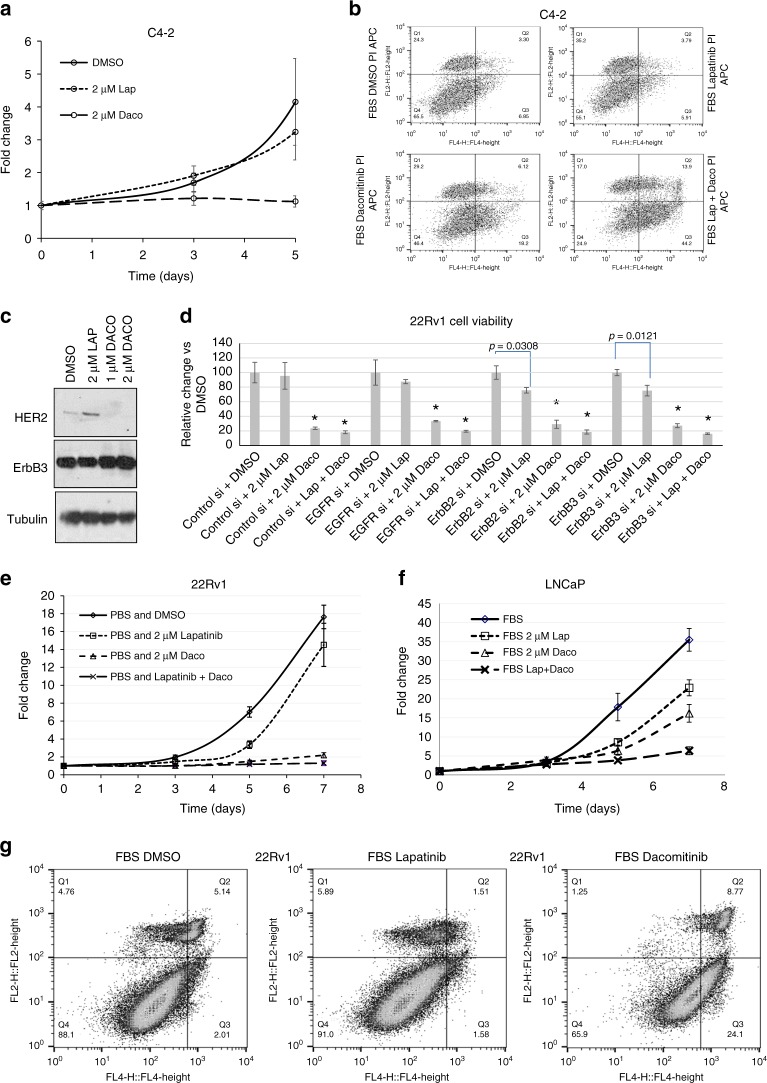
Dacomitinib overcomes resistance in CRPC cells by preventing lapatinib-like increases in HER2. **a** MTT assay showing that castration-resistant C4-2 cells are extremely sensitive to low concentrations of dacomitinib compared to lapatinib. Each point represents mean ± S.D. of three biological replicates. **b** Flow cytometry demonstrating that C4-2 cells show increased Annexin V-marked apoptosis (Q3) when treated with dacomitinib and this is greatly increased with a combination of lapatinib and dacomitinib. **c** Western blots showing that C4-2 cells exhibit increased HER2 protein, but not ErbB3, in response to lapatinib but not dacomitinib. **d** 22Rv1 cells were subjected to control siRNA as well as EGFR, HER2 or ErbB3 siRNA, and then further treated with DMSO, 2 µM lapatinib, 2 µM dacomitinib or a combination thereof. MTT assay showed that knockdown of HER2 and ErbB3, but not EGFR, sensitised 22Rv1 cells to lapatinib. Dacomitinib greatly decreases cell viability in 22Rv1 cells but the combination with lapatinib has no additional effect. Experiments were performed in triplicate; all data is shown relative to the corresponding mean of DMSO treated cells for each siRNA used (of mean ± S.D. of three biological replicates), in order to emphasise relative differences (*p < 0.0001). **e** MTT assay over 0–7 days to show that 22Rv1 is extremely sensitive and displays greatly-decreased viability to low concentrations of the irreversible EGFR inhibitor dacomitinib (‘Daco’). The addition of lapatinib does not enhance dacomitinib’s effect. **f** High-passage LNCaP cells treated similarly do not demonstrate such a dramatic effect in response to single-agent dacomitinib presumably because they are still not as reliant on EGFR/HER2-mediated signalling as the far-more aggressive 22Rv1 cell line. In contrast however, the combination of lapatinib and dacomitinib displays a synergistic effect in these cells. For both **e** and **f**, each point represents mean ± S.D. of three biological replicates. **g** Flow cytometry in live cells dually stained with propidium iodide and Annexin V show that dacomitinib greatly increases apoptosis in 22Rv1 cells. Lapatinib and dacomitinib were used at 2uM and dissolved in 100% DMSO. Twenty thousands events were analysed per condition

Since both HER2 and ErbB3 contributed to lapatinib resistance in C4-2 cells **(**Fig. 3c**)**, we compared the effects of dacomitinib, an FDA-approved, irreversible pan-ErbB inhibitor to lapatinib (Table S5). In C4-2- cells, at 2 uM, lapatinib did not have a significant effect on cell growth over 5 days (*p* = 0.0849) whereas dacomitinib (over the same time period) successfully inhibited cell viability by 89.8% (*p* = 0.0103) (Fig. 4a). This difference is caused by a 3-fold increase in apoptosis in cells treated for 72 h with dacomitinib (18.2%) compared to vehicle (6.85%) or lapatinib (5.91%). Combination treatment with dacomitinib and lapatinib synergised to greatly increase apoptosis (44.2%) (Fig. 4b). While lapatinib increased HER2 levels, dacomitinib prevented this effect but, like lapatinib, did not affect ErbB3 levels (Fig. 4c).

To investigate the effect of RTKs on dacomitinib sensitivity, we silenced EGFR, HER2 or ErbB3 in 22Rv1 cells prior to treatment with lapatinib or dacomitinib (Fig. 4d). Similar to C4-2, knockdown of ErbB3 and HER2, but not EGFR, sensitised 22Rv1 cells to lapatinib; in addition, dacomitinib also suppressed viability of 22Rv1 cells, alone or together with lapatinib (Fig. 4d), and in C4-2 cells (not shown). Efficacy of receptor knockdown is shown in Fig. S4. Similar to C4-2, in 22Rv1 cells, where lapatinib induced a 17% decrease in viability (*p* = 0.1295), dacomitinib caused an 87.6% decrease in viability (*p* < 0.0001) (Fig. 4e). LNCaP cells were not as responsive to dacomitinib (35.5% loss of viability with lapatinib, *p* = 0.0055; 54.4% loss with dacomitinib, *p* = 0.0012), indicating specificity for CRPC cells (Fig. 4f). Similar to C4-2 cells, 22Rv1 cells underwent dacomitinib-induced apoptosis (24.1%), in contrast to lapatinib (1.58%), which was comparable to controls (2.01%) (Fig. 4g). Taken together, these results demonstrated that dacomitinib did not increase HER2 levels and reduced cell viability by inducing apoptosis in CRPC cell lines.

### Dacomitinib, but not lapatinib, prevents EGFR/HER2 heterodimerisation and downstream signalling of EGFR family members in CRPC cells

**Fig. 5:**
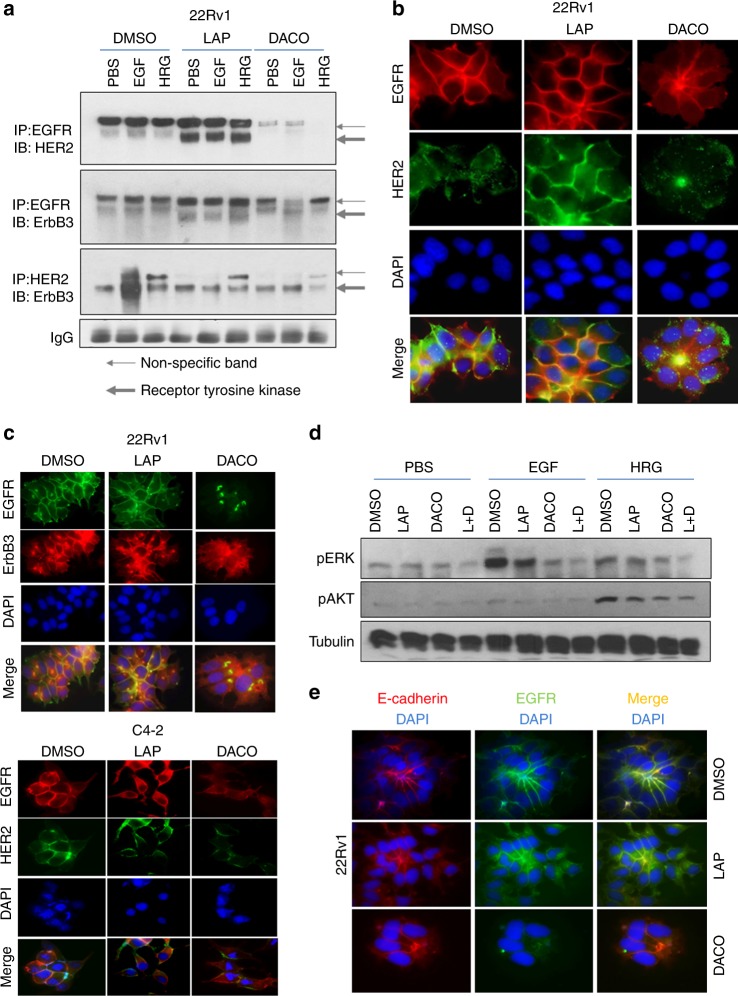
Dacomitinib, unlike lapatinib, disrupts membrane localisation, decreases EGFR-HER2 dimerisation and downstream signalling of EGFR family members in CRPC cells. **a** In 22Rv1 cells, pulldown assays demonstrate the significant disappearance of EGFR-HER2 dimers with dacomitinib and, to a lesser extent, HER2-ErbB3 dimers. Cells were cultured in the presence of DMSO, 2 µM lapatinib or 2 µM dacomitinib for 72 h, at the end of that period, they were further treated with PBS, 10 ng/ml EGF or 50 ng/ml HRG for 30 min. Note that in these cells, an upper non-specific band (narrow arrow) appeared in the immunoprecipitated lysates in addition to the RTK band (thick arrow) that was not apparent in regular Westerns. **b** Immunofluorescent microscopy indicating the loss of EGFR and HER2 staining in dacomitinib but not lapatinib-treated cells. 22Rv1 cells were treated with DMSO, 2 µM lapatinib or 2 µM dacomitinib for 72 h, then fixed and stained with anti-EGFR antibody (secondary—Rhodamine-tagged) and anti-HER2 (secondary—FITC tagged) as well as DAPI (blue). Merged pictures depict colocalisation. **c** 22Rv1 (upper) or C4-2 (bottom) cells were cultured in androgen-containing media and treated with 2uM lapatinib or dacomitinib for 72 h. Hundred percent DMSO was used as the control. (upper) Lapatinib treatment results in prominent EGFR and ErbB3 membrane staining. Dacomitinib treatment results in the disappearance of EGFR and ErbB3 protein. Cell numbers also appeared greatly reduced. (bottom) Dacomitinib also decreases the staining intensity of HER2 and EGFR in C4-2 cells. **d** Western blots comparing the effects of dacomitinib and lapatinib on phosphorylated p44/42 ERK1/2 (Thr202/Tyr204) and phosphorylated AKT (Ser473). **e** Immunofluorescent imaging showing that dacomitinib alters EGFR membrane staining but does not disrupt the membrane itself. Cells were stained with anti-E-cadherin (rhodamine tagged) and EGFR (FITC tagged). In control cells, EGFR colocalised with E-cadherin (yellow merge) showing that both proteins were expressed in the plasma membrane. Upon treatment with 2 µM lapatinib, the colocalisation intensified, showing increased localisation of EGFR in the membrane. On the other hand, 2 µM dacomitinib decreased EGFR levels in the membrane but left E-cadherin levels intact, demonstrating that membrane integrity was not compromised. Pictures were magnified at ×100

Since EGFR/HER2 heterodimer levels were increased by lapatinib, we investigated whether dacomitinib behaved similarly (Fig. 5a). As in C4-2 cells, in 22Rv1, lapatinib significantly increased EGFR/HER2 dimers but dacomitinib decreased their dimerisation. On the other hand, EGFR/ErbB3 and HER2/ErbB3 heterodimers were relatively unaffected by both lapatinib and dacomitinib (Fig. 5a). It is therefore likely that the mechanism of dacomitinib efficacy stems from its ability to prevent EGFR/HER2 dimerisation. Since receptor heterodimerisation occurs at the plasma membrane we investigated whether dacomitinib prevented EGFR/HER2 formation by affecting their membrane localisation. Lapatinib enhanced HER2 (but not EGFR) membrane localisation (Fig. 5b); whereas dacomitinib eliminated membrane localisation of both EGFR and HER2 in 22Rv1 cells (Fig. 5b). Similarly, dacomitinib prevented HER2/ErbB3 colocalisation (Fig. S5A). Similar effects were observed in C4-2 cells, where EGFR/HER2 colocalisation was disrupted by dacomitinib but enhanced by lapatinib (Fig. 5c, lower).

Investigating the downstream targets of these dimers showed that EGF-induced, EGFR-activated ERK1/2 phosphorylation was significantly decreased by dacomitinib but not lapatinib (Fig. 5d). On the other hand, AKT phosphorylation was significantly induced by heregulin-1β and was inhibited by both drugs (Fig. 5d). This suggested that ERK phosphorylation, downstream from EGFR/HER2 heterodimerisation, differentiated the effects of dacomitinib from those of lapatinib, and that decreased EGFR/HER2 heterodimerisation likely resulted from lack of EGFR/HER2 membrane localisation in the presence of dacomitinib. In support of the lack of a role for AKT in lapatinib-induced growth in vitro shown above, immunoblot analyses of mouse tumours showed that in CWR22 tumours, lapatinib suppressed AKT phosphorylation at S473, whereas ERK phosphorylation at T202/Y204 was not altered (Fig. S5B). We noted with interest that the disruption of membrane localisation of EGFR or HER2 preserved membrane integrity (and perhaps membrane protein in general) since the levels of E-Cadherin in the cells continued to be consistent (although low) despite lapatinib or dacomitinib treatment (Fig. 5e, Fig. S5C).

### Lapatinib increased HER2 levels by promoting protein synthesis but dacomitinib did not have a similar effect

**Fig. 6:**
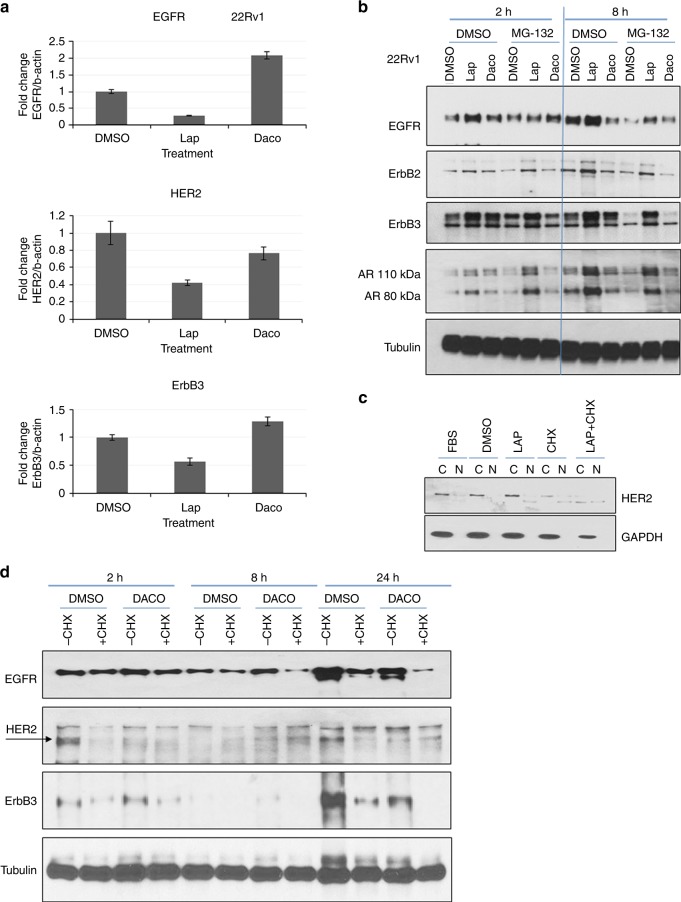
Lapatinib increased HER2 levels by elevating protein synthesis while dacomitinib prevented this effect. **a** qPCR showing the effects of lapatinib on EGFR, HER2 or ErbB3 mRNA. 22Rv1 cells were cultured in serum-containing media with 2 µM lapatinib or dacomitinib before being lysed with TRIZOL reagent as per the manufacturer’s instructions. Results represent mean ± S.D. of experiments performed in triplicate biological replicates. **b** 22Rv1 cells were treated for 2 or 8 h with vehicle or 2 µM lapatinib with or without 5 µg/ml MG-132 and fractionated as described. **c** Cells were pre-treated for 3 h with 100 µg/ml of cycloheximide before being washed off and allowed to recover for 24 h in fresh media containing either vehicle (DMSO) or 2 µM lapatinib. Fractionation was performed as detailed. **d** Dacomitinib decreases protein stabilities of EGFR, HER2 and ErbB3 despite causing an increase in EGFR and ErbB3 mRNA. Cells were plated and allowed to attach overnight at 37 °C before being pre-treated for 3 h with 100 µg/ml of cycloheximide dissolved in 100% DMSO or an equivalent volume of 100% DMSO. Cells were subsequently washed twice with 1X phosphate-buffered saline (PBS) and allowed to recover in fresh media containing either 2uM dacomitinib or an equivalent volume of 100% DMSO. Cells were collected, and lysates prepared at 2, 8 or 24 h for analysis by western blotting

Finally, we investigated possible mechanisms for increased EGFR and/or HER2 levels seen with lapatinib but not dacomitinib treatment. qRT-PCR analyses demonstrated that in 22Rv1 cells, lapatinib suppressed EGFR, HER2 and ErbB3 mRNA indicating that transcriptional regulation was not a cause of HER2 accumulation (Fig. 6a). Similar effects were observed in C4-2 cells, where change in mRNA levels were insignificant (Fig. S6A). On the other hand, dacomitinib stimulated mRNA levels of EGFR in 22Rv1 cells, although its effects on HER2 and ErbB3 mRNA were not significant. Despite this, protein levels of HER2 increased significantly with lapatinib (Fig. 6b). Therefore, we investigated whether changes in translational or post-translational mechanisms led to an increase in HER2 levels with lapatinib treatment. HER2 expression is regulated by c-Cbl, an ubiquitin ligase^29,30^ that degrade it and therefore control its levels. We investigated whether reduced proteasomal activity caused accumulation of HER2 following lapatinib treatment. Hence, we simulated these conditions using the reversible proteasome inhibitor MG-132 in the presence or absence of lapatinib or dacomitinib. Twenty-four hour culture in the presence or absence of 2 µM lapatinib, dacomitinib or 5 µM MG-132 or a combination of both showed that MG-132 treatment reduced RTK accumulation by lapatinib, thereby indicating that proteasomal activity was responsible for HER2 accumulation upon lapatinib treatment. (Fig. 6b). MG-132 synergised with dacomitinib to further reduce HER2 and ErbB3—hence it is likely that dacomitinib did not affect the proteasomal pathway. Similarly, AR levels were also affected by lapatinib, but not dacomitinib; however, MG-132 had no effect on AR levels.

Since HER2 mRNA levels are reduced by lapatinib; however, one way that a significant increase in protein levels occurs is by an increase in protein synthesis. Hence, we used the protein synthesis inhibitor cycloheximide to determine the effect of lapatinib on HER2 synthesis. The increase in HER2 expression by lapatinib (Fig. 6c) was suppressed by cycloheximide, indicating that lapatinib-induced increases in protein levels were translationally regulated. Under control conditions, RTK protein levels increased over a period of 24 h, but cycloheximide prevented this effect by suppressing protein synthesis both in the presence or absence of dacomitinib, both in C4-2 (Fig. 6d) and in 22Rv1 cells (Fig. S6B). Taken together, these results demonstrated that lapatinib increased HER2 levels by increasing protein synthesis, an effect that was not observed with dacomitinib treatment.

## Discussion

Multiple reports have described the involvement of the EGFR family of RTKs in prostate tumour development and progression.^9,31–34^ EGFR and HER2 have long been studied as targets for anti-cancer therapy.^35–37^ Lapatinib has been a standard-of-care for women with HER2-positive breast cancer for several years.^19^ Preclinical studies have indicated a significant effect of HER2 in CRPC;^38,39^ and lapatinib was reported to prevent abiraterone acetate and enzalutamide resistance in LAPC-4 and LNCaP tumours, respectively.^38,40^ However, in clinical trials, lapatinib failed to effectively suppress CRPC progression^20^ and did not affect HSPC patients either.^21,22^ In the past, we had predicted that simultaneous inhibition of multiple ErbB receptors would benefit patients with CRPC.^18^ In recent times, a number of pan-ErbB inhibitors have been developed, such as afatinib,^41^ neratinib^42^ and dacomitinib.^23^ In this paper, we investigated why PCa cells may be resistant to lapatinib and whether a pan-ErbB inhibitor may be more effective in preventing PCa progression.

In this paper, we used the CWR22/22Rv1 mouse model of hormone-sensitive and castration-resistant prostate cancer that more accurately reflected the effects of lapatinib in human patients. In this model, lapatinib at physiological doses failed to affect growth of xenograft tumours in either intact or in castrated mice. Investigation of the mechanism of resistance showed that remarkably, lapatinib increased HER2 levels in the tumours. Based on these results, we investigated whether the same is seen in human patients when treated with lapatinib. Utilising serum samples from a clinical trial where lapatinib was used in CRPC patients,^20^ we determined that, similar to the animal model, 92.3% of the patients experienced an increase in serum levels of HER2. Additional in vitro analyses using physiological levels of lapatinib demonstrated this drug’s inability to significantly decrease cell viability; however, it increased HER2 protein and EGFR/HER2 heterodimers in CRPC lines. Knockdown of HER2 sensitised CRPC cells to lapatinib, indicating that the observed resistance to this drug is mediated by the increase in this RTK.

It may be noted that the only specimen available from the patients who had participated in the lapatinib trial^20^ are serum samples. While EGFR and HER2 levels in these samples can come from anywhere in the body, multiple studies in both animal models and in patients with various types of cancers have demonstrated that in patients with cancer, serum HER2 levels accurately reflect the intratumoral levels of this protein.^43–45^ In metastatic prostate cancer, circulating levels of HER2 have often been used as a predictive marker of progression.^46–49^ Therefore, we are assured that the effects of lapatinib observed in the serum also reflect the effects of lapatinib occurring in the tumour tissue.

Since CRPC cell viability is affected by HER2, we investigated whether a pan-ErbB inhibitor would be more effective in CRPC than lapatinib. Dacomitinib is a second-generation TKI that has recently gained FDA-approval for first-line treatment of patients with metastatic non-small cell lung cancer (NSCLC) with specific EGFR mutations.^24^ This TKI has not previously been reported on in PCa. Dacomitinib is very different structurally and functionally from lapatinib, and key differences between the two drugs are presented in Table S5. At comparable equimolar concentrations, dacomitinib significantly decreased cell viability and increased apoptosis in CRPC cells, whereas lapatinib failed to have these effects. Significantly, dacomitinib was more effective in CRPC compared to HSPC, which makes it a selective drug for the more advanced patients. Dacomitinib, but not lapatinib, decreased ErbB heterodimer formation and prevented plasma membrane localisation of EGFR and HER2 while preserving membrane integrity. Although EGFR alone did not affect lapatinib efficacy, it is the presence of the EGFR/HER2 dimers that influenced the action of both drugs. Our data suggest that in CRPC, HER2 is present at relatively low levels. This is reflected in low serum levels of HER2 in sera and low HER2 levels in xenograft tumours. Despite low initial levels of HER2, a substantial increase, such as that seen upon lapatinib treatment, can enhance proliferation. The low initial levels of HER2, together with the increase, can therefore operate as a rate-limiting step in ways that EGFR, which is abundant, cannot. However, dacomitinib can prevent not only HER2 kinase activity, but also the formation of EGFR/HER2 dimers, likely by preventing receptor membrane localisation. These preclinical data support the hypothesis that pan-ErbB family inhibitors like dacomitinib, or neratinib may be viable therapeutic strategies for CRPC patients and strengthen the case for revisiting the emphasis on these TKIs.

Additionally, we observed that the mechanism by which lapatinib raised HER2 levels was by decreased proteasomal degradation and increased protein synthesis (not increased gene transcription), which is not observed with dacomitinib. Others have reported that lapatinib stabilised HER2 levels and its accumulation on the cell surface in breast cancer.^50^ They report enhanced EGFR/HER2 and HER2/ErbB3 dimer formation with lapatinib but conclude that the dimers will be inactive. Here we demonstrate that in our cells, conditions that resulted in dimer formation also transmit signals to downstream targets—specifically ERK phosphorylation, and hence the dimers are active. Interestingly, Akt phosphorylation was affected by both drugs equally, and therefore, does not appear to play a significant role in lapatinib resistance. Our results imply that increased HER2 causes resistance to lapatinib mainly because it is a rate-limiting step in the formation of EGFR/HER2 dimers. On the other hand, dacomitinib prevents the formation of these dimers by inhibition of their accumulation in the membrane.

In summary, we have demonstrated that failure of lapatinib in clinical trials of CRPC stems from its ability to increase HER2 levels significantly (although it also increased EGFR in some cases, the effect was not shown to be statistically significant). The mechanism of this increase is via enhanced protein synthesis rates, resulting in accumulation of excess HER2 in the plasma membrane, formation of EGFR/HER2 dimers and transmission of signals to downstream targets that prevent loss of cell viability. On the other hand, dacomitinib prevented EGFR and HER2 (and ErbB3) membrane accumulation, thereby preventing EGFR/HER2 dimerisation, which inhibited downstream signalling and resulted in cell death. This mechanism is described in Fig. S7. In PCa cells, HER2 may be a relatively unsuitable target because it did not undergo gene amplification and increased protein expression,^51–53^ hence inhibitors that specifically target HER2 may not be effective, despite the significant role of HER2 in this process. Further studies will indicate whether suppression of HER2 transcription by lapatinib leads to activation of a feedback loop that resulted in enhanced protein synthesis, but these studies are beyond the scope of the current project. Data reported here, therefore, demonstrate that despite initial failure of inhibitors of the EGFR family of RTKs in CRPC, new generations of such inhibitors that target all members of this family are more likely to succeed.

## Supplementary information

Supplemental Materials

## Data Availability

All data generated or analysed during this study are included in this published article [and its supplementary information files]. Additional data and methods are described in Supplementary information available at the British Journal of Cancer’s website
