# Supplementary material for: Dacomitinib, but not lapatinib, suppressed progression in castration-resistant prostate cancer models by preventing HER2 increase
Source: Br J Cancer. 2019 Jun 18;121(3):237–48. doi: 10.1038/s41416-019-0496-4 (PMC6738116; doi:10.1038/s41416-019-0496-4)
Supplement: Supplementary file 1 — Supplemental Materials [file 41416_2019_496_MOESM1_ESM.pdf]

SUPPLEMENTARY MATERIAL

Dacomitinib, unlike lapatinib, suppressed progression in castration resistant prostate cancer models by preventing HER2 increase

Table S1: Patient Characteristics

Characteristics of 14 patients whose sera were available pre- and post-treatment for EGFR and HER2 evaluation of 29 patients who participated in a Phase 2 clinical trial reported earlier [1] -

number of patients	14
Age [median (range)]	79 (61-91)
HgB [median (range)]	13.1 (11-14.6)
Pre-treatment PSA [median (range)]	17.55 (4.5 – 196.5)
Alk Phos [median (range)]	83 (59 – 104)
Measurable disease	8 (57.14%)
Bone disease	4 (28.57%)
Prostatectomy	7 (50.00%)
%increase in PSA [median (range)]	63.9 (31.5 – 128)
Time to Progression [median (range)]	28 (27 – 476)

Table S2: Molecular Characteristics of Prostate Cancer Model Systems

Molecular Characteristics of Cell Lines Used in this Study

**Cell Line**	**Origin**	**AR Status**	**IC_{50} (Enzalutamide)**	**Sensitivity to enzalutamide**	**SPOP**	**ETS fusions**
**CWR-R1**	Relapsed CWR22	H874Y, AR splice variants	>10 µM	Resistant	NA	NA
**CWR22Rv1**	Relapsed CWR22	H874Y, exon 3 dup, AR splicing	>10 µM	Resistant	WT [2]	High levels of ETV1 protein [3]
**C4-2**	Relapsed LNCaP	T877A, overexpression	3.5 µM	Sensitive	WT [4]	Lacks TMPRSS2-ERG [5] but possesses TMPRSS-ETV1 expression [6]
**C4-2B**	Relapsed LNCaP	T877A, overexpression	3 µM	Sensitive		TMPRSS-ETV1 expression [6]
**LNCaP**	Primary tumor	T877A	2 µM	Sensitive	WT [4]	Lacks TMPRSS2-ERG [5] but possesses TMPRSS-ETV1 expression [6]

Table S3: Primer sequences used

Target	Forward	Reverse
**EGFR**	5' AACACCCTGGTCTGGAAGTACG 3’	5' TCGTTGGACAGCCTTCAAGACC 3’
**HER2/ErbB2**	5' AGCATGTCCAGGTGGGTCT 3’	5' CTCCCTCCTCGCCCTCTTG 3’
**HER3/ErbB3**	5’ CCAGGTCTACGATGGGAAGT 3’	5’ CTGCCATTGTCCTTCACCAC 3’

Table S4: siRNA SMARTPool target sequences

Target	Target Sequences 5’ – 3’
**EGFR**	1. CCGCAAAUUCCGAGACGAA<br>2. CAAAGUGUGUAACGGAAUA<br>3. GUAACAAGCUCACGCAGUU<br>4. GAGGAAAUAUGUACUACGA
**HER2/ErbB2**	1. GGACGAAUUCUGCACAAUG<br>2. GACGAAUUCUGCACAAUGG<br>3. CUACAACACAGACACGUUU<br>4. AGACGAAGCAUACGUGAUG
**HER3/ErbB3**	1. GCAGUGGAUUCGAGAAGUG<br>2. AGAUUGUGCUCACGGGACA<br>3. GUGGAUUCGAGAAGUGACA<br>4. GCGAUGCUGAGAACCAAUA
**Control (scrambled)**	1. UAGCGACUAAACACAUCAA<br>2. UAAGGCUAUGAAGAGAUAC<br>3. AUGUAUUGGCCUGUAUUAG<br>4. AUGAACGUGAAUUGCUCAA

Table S5: Comparison of lapatinib vs dacomitinib

Characteristics of the Two Main Drugs Used in this Study

**Lapatinib**	**Dacomitinib**
Reversible tyrosine kinase inhibitor (TKI)	Irreversible tyrosine kinase inhibitor (TKI)
ErbB2/EGFR inhibitor	pan-ErbB inhibitor
Prescribed dose =1500mg daily	Prescribed dose =45mg daily
Induces cell cycle arrest but not much apoptosis	Induces apoptosis and cell cycle arrest
FDA-approved for breast cancer	FDA-approved for lung cancer
Median IC_{50} (human cells)=4 μM	Median IC_{50} (human cells)=0.5 μM
Modest decrease in protein levels of ErbB dimers	Strong decrease in protein levels of ErbB dimers

SUPPLEMENTARY FIGURES:

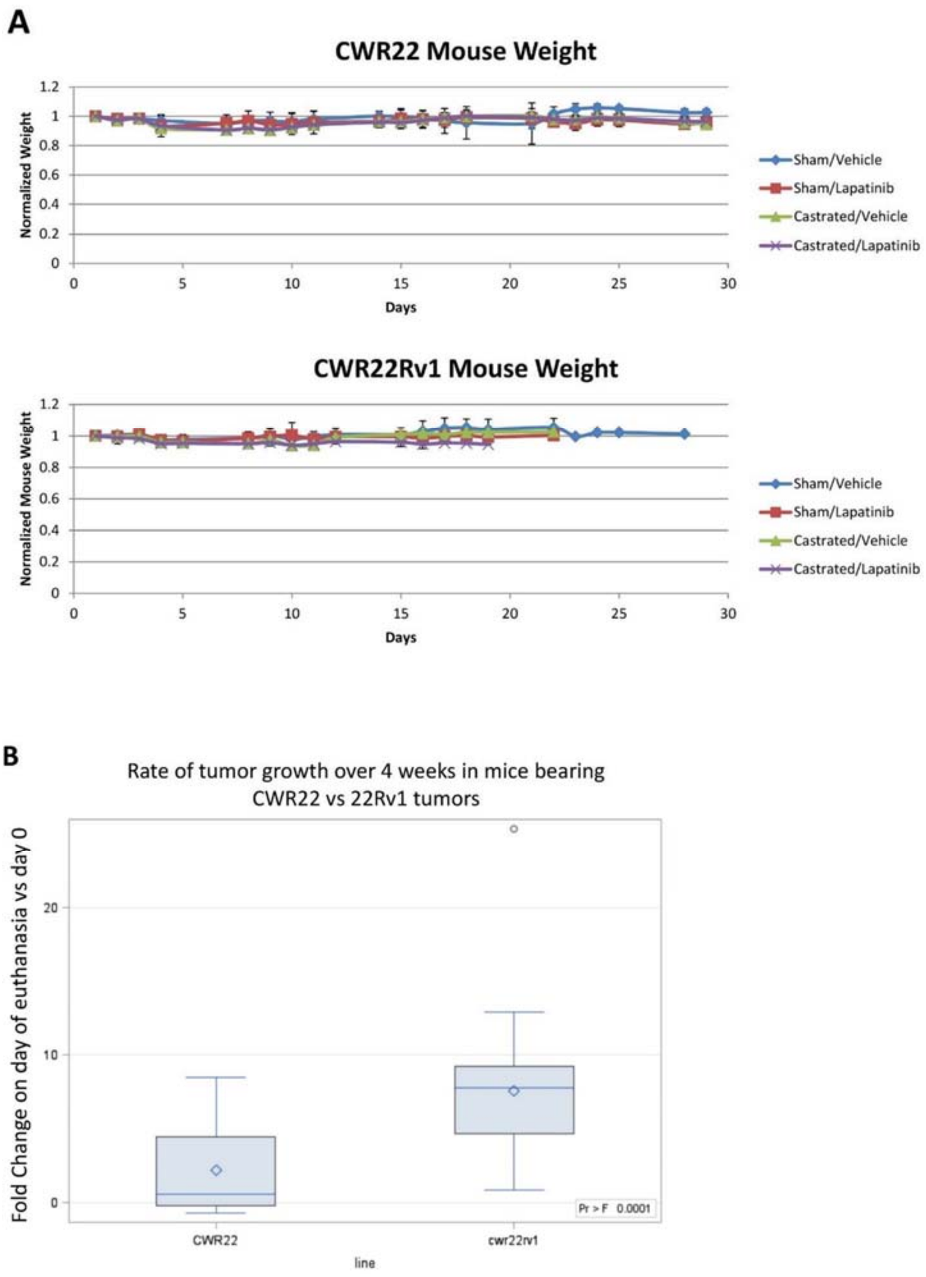

FIGURE S1. Analysis of body weights and tumor sizes of mice bearing human tumor derived CWR22 and 22Rv1 tumor lines.

(A) Graphical representation of body weights of all mice bearing CWR22 and CWR22-Rv1 xenografts demonstrating the feasible safety profile of lapatinib. Lapatinib was administered at 100mg/kg of body weight per mouse. **(B)** Comparison of rate of tumor growth in mice bearing CWR22 and 22Rv1 tumors. Rate of growth was calculated based on tumor size at days 2-28 over tumor size at day 1. Negative fold change denoted tumor regression.

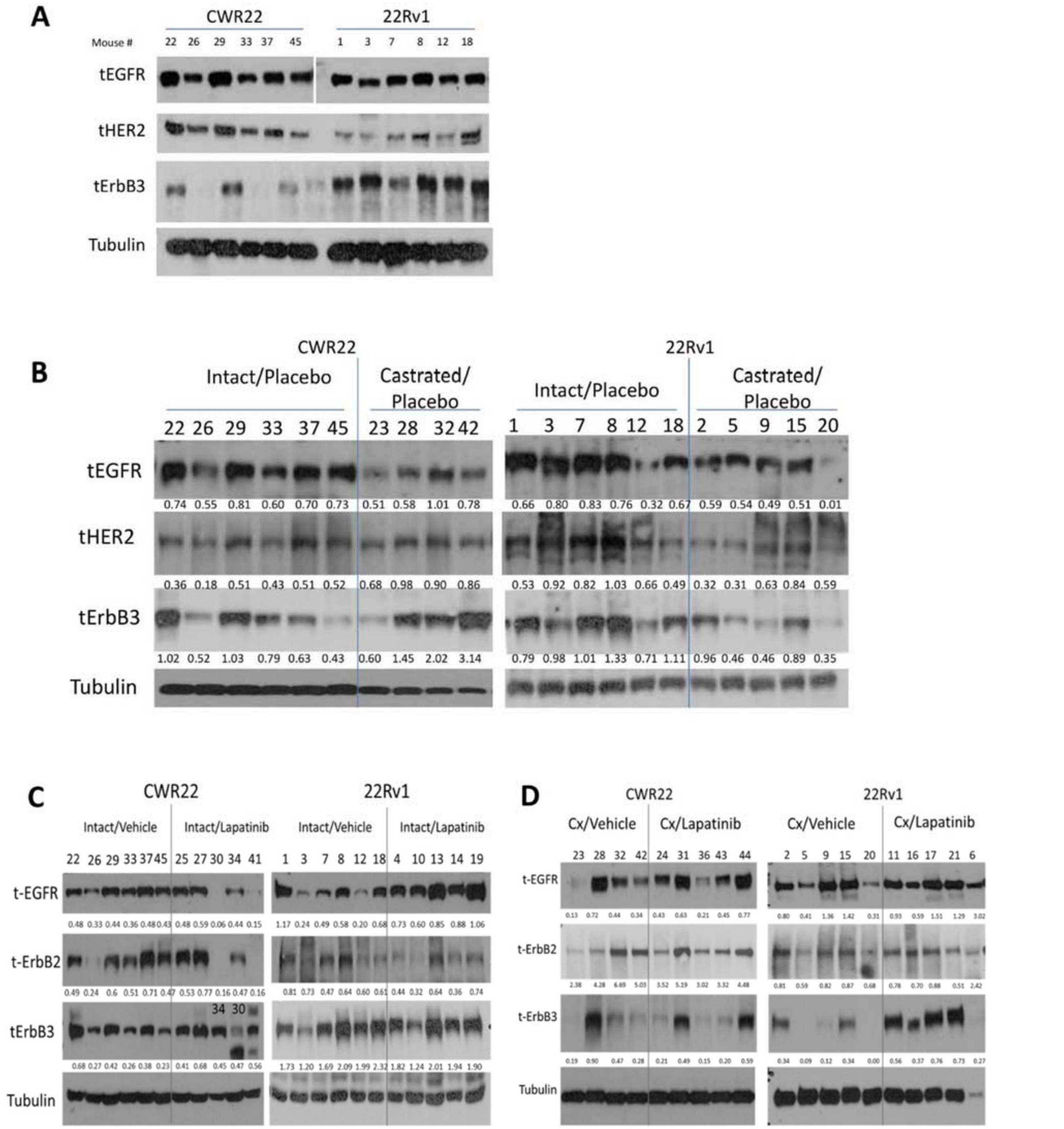

FIGURE S2. Analysis of tumor lysates from immunodeficient mice bearing CWR22 and 22Rv1 tumors. **(A)** Comparison of EGFR, HER2 and ErbB3 levels in lysates from Hormone-sensitive (HS) CWR22 xenografts compared to castration-resistant prostate cancer (CRPC) xenografts CWR22Rv1 in immunocompromised mice. There were 6 mice in each group. HER2 protein was difficult to detect in 22Rv1 xenografts. This is in agreement with the general difficulty of observing HER2 protein in human prostate tumor tissue [1]. **(B)** Western blots for comparison of RTK expression in tumor lysates from tumors in mice bearing CWR22 or 22Rv1 tumor xenografts who were left intact or were subjected to bilateral castration. **(C)** Western blots for comparison of EGFR, HER2 and ErbB3 levels in tumor lysates from mice bearing CWR22 and 22Rv1 xenograft tumors who were treated with vehicle or lapatinib. The mice had further been left intact or castrated.

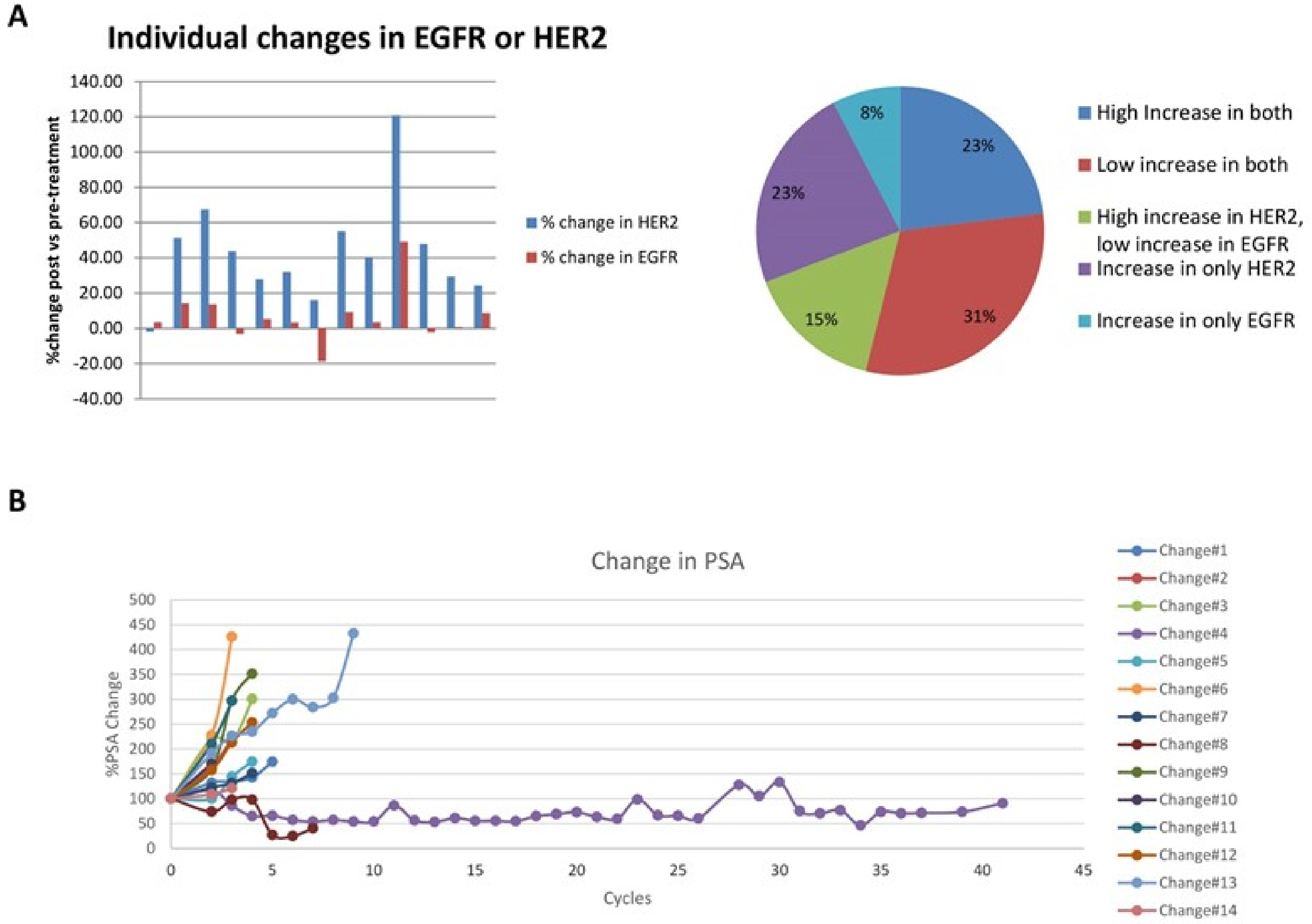

FIGURE S3: Analysis of serum samples from patients with castration resistant prostate cancer treated with Lapatinib for a Phase II open label single arm clinical trial.

(A, left) Bar graph representing percentage changes in serum EGFR and HER2 levels in lapatinib-treated patients. Each set represents a single patient. **(A, right)** Pie chart showing percentages of lapatinib-treated patients who displayed elevated either serum EGFR or HER2 or both **(B)** Percentage changes in serum PSA as measured by ELISA over 10 cycles of treatment for each one of 14 patients enrolled in a Phase II, open-label, single-arm trial to test the efficacy of lapatinib. Note the prolonged and durable response to lapatinib as evidenced by stable serum PSA for Patient #4.

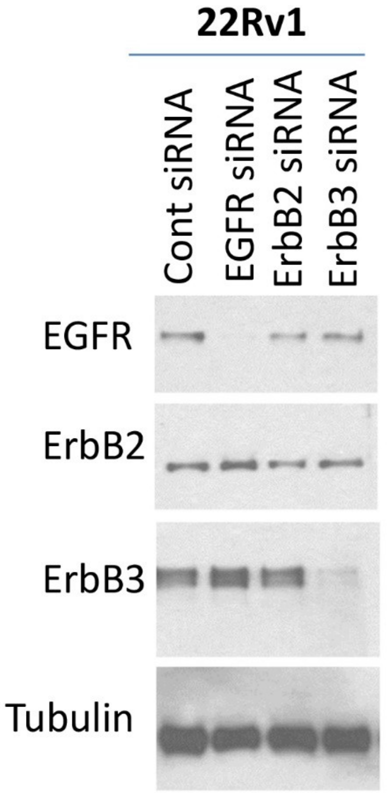

FIGURE S4. Immunoblot verifying EGFR family silencing in 22Rv1 cells.
22Rv1 cells were plated at semi-confluency and after 24 hours, subjected to RNA inhibition with EGFR, HER2 and ErbB3 siRNA. After 72 hours, cells were collected, lysed and run on SDS-PAGE. Membranes after transfer were subjected to staining with anti-RTK antibodies.

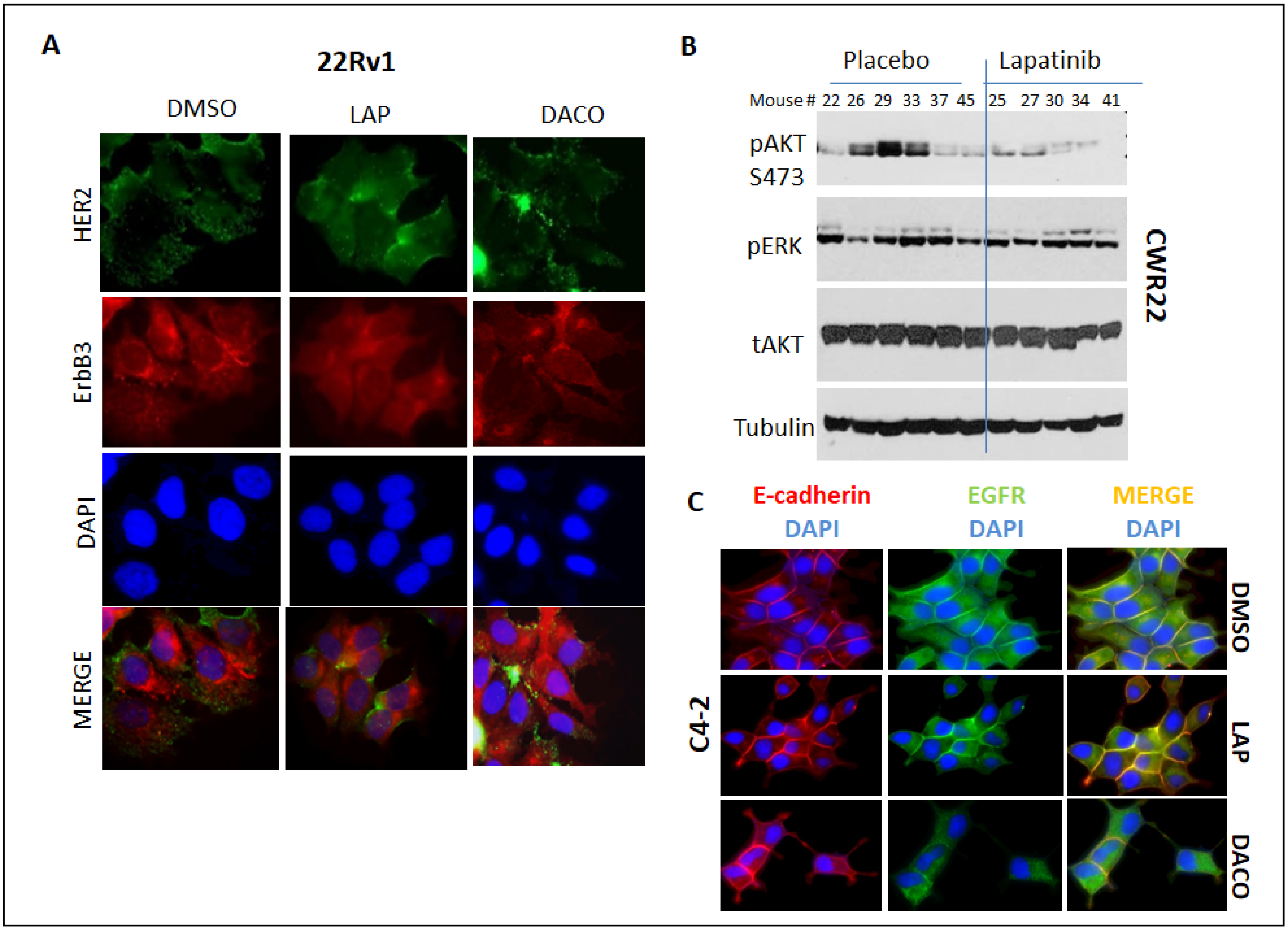

FIGURE S5. Comparison of the effects of dacomitinib and lapatinib on localization, and downstream signaling of EGFR family members.

(A) (B) Immunofluorescent microscopy indicating the loss of HER2 and ErbB3 staining in dacomitinib but not lapatinib-treated cells. 22Rv1 cells were treated with DMSO, 2 µM lapatnib or 2 µM dacomitinib for 72 hours, then fixed and stained with anti-ErbB3 antibody (secondary – Rhodamine-tagged) and anti-HER2 (secondary - FITC tagged) as well as DAPI (blue). . **(B)** Lapatinib modestly decrease AKT but not ERK signaling in HS xenografts. Immunoblots compared Akt and ERK phosphorylation levels in lysates from CWR22 tumor xenografts in intact immunocompromised mice treated with placebo or lapatinib as described. Tumors were removed when the animals were anesthetized. There were 6 mice in each group. **(C)** C4-2 cells display decreased co-localization of EGFR and E-Cadherin in the presence of dacomitinib but not lapatinib. Immunofluorescent images are taken at high-magnification (100X). Note that dacomitinib leaves membrane integrity intact.

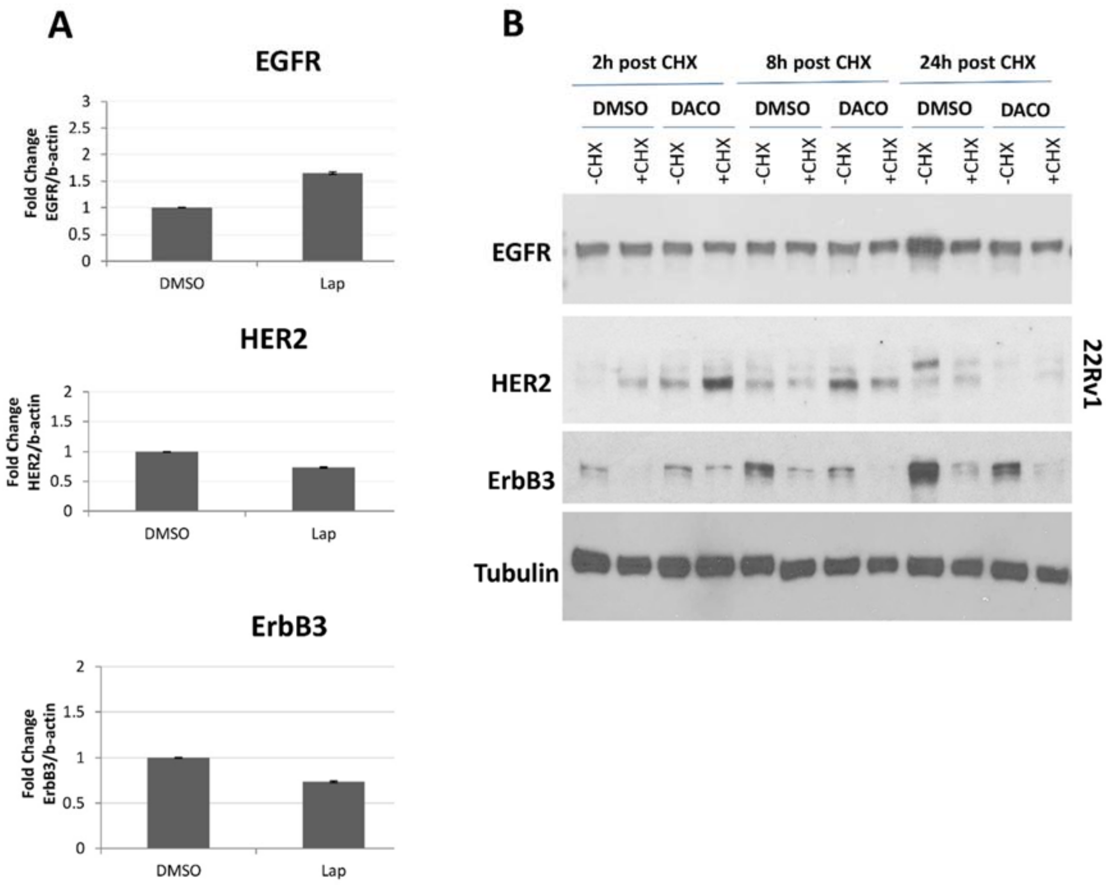

FIGURE S6. Lapatinib effect on EGFR and HER2 mRNA levels and the effect on protein synthesis by dacomitinib.

(A) qPCR showing that in C4-2 cells, lapatinib has no significant effect on EGFR, HER2 and ErbB3 mRNA. Cells were cultured in serum-containing media with 2µM lapatinib or dacomitinib prior to lysing with TRIZOL reagent as per the manufacturer's instructions. Results represent an average±S.D. of experiments performed in triplicate biological replicates. **(B)** Dacomitinib decreases stability of EGFR, HER2 and ErbB3 protein despite . Cells were plated and allowed to attach overnight at 37º C prior to pre-treatment for 3h with 100µg/ml cycloheximide dissolved in DMSO or an equivalent volume of DMSO. Cells were subsequently washed twice with 1X phosphate-buffered saline (PBS) and allowed to recover in fresh media containing either 2µM dacomitinib or an equivalent volume of DMSO. Cells were collected and lysates prepared at 2, 8 or 24h for analysis by western blotting.

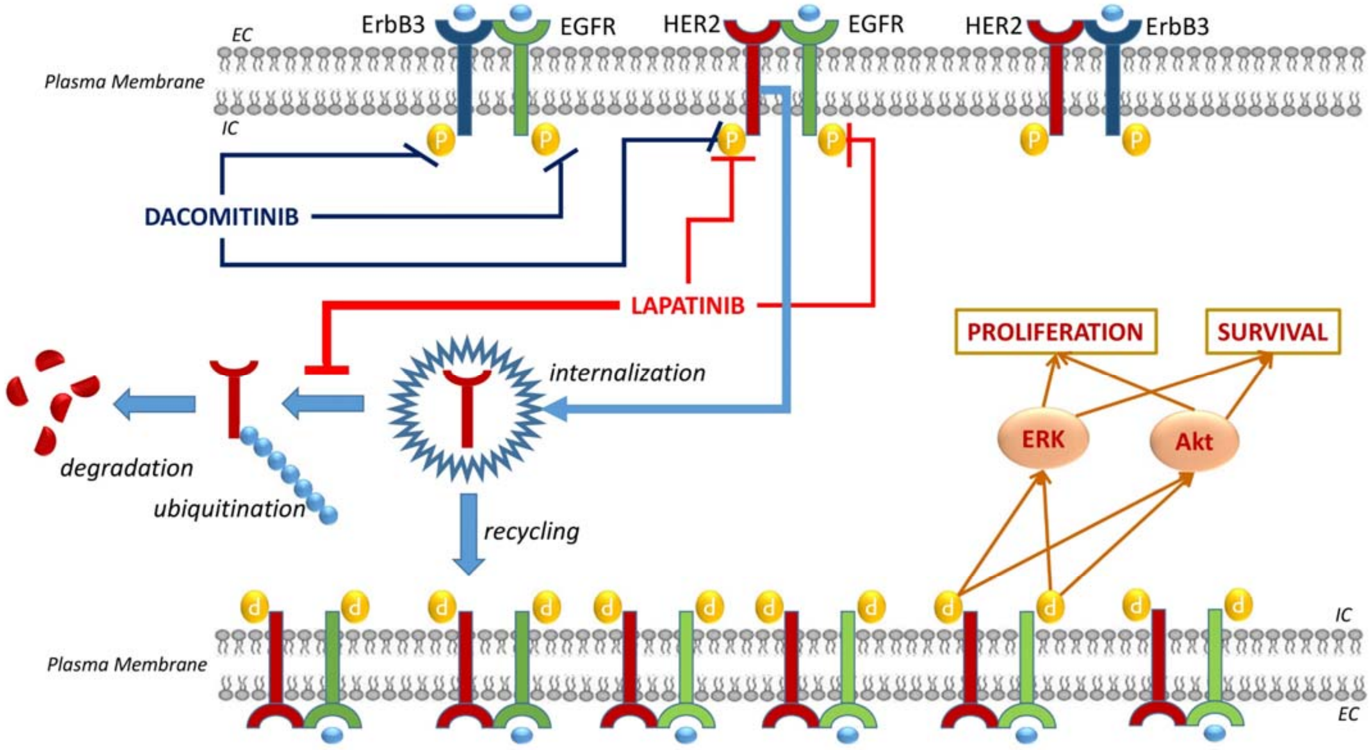

FIGURE S7. Mechanism for dacomitinib efficacy in prostate tumor cells. In untreated prostate cancer cells, receptor tyrosine kinases (RTK) including HER2 is internalized to a cytoplasmic compartment following activation. Internalized RTKs are then either ubiquitinated and undergo intracellular (IC) degradation, or is recycled back to the plasma membrane (PM). Lapatinib treatment impairs RTK degradation, resulting in accumulation of HER2 on the plasma membrane. This enables an increase in EGFR/HER2 heterodimers following EGFR ligand binding in the extracellular (EC) space, which signal to the ERK and Akt pathways leading to continued tumor cell proliferation and survival. In contrast, dacomitinib does *not* prevent HER2 degradation, thereby averting HER2 membrane accumulation, resulting in loss of EGFR/HER2 heterodimerization, inhibiting Akt and ERK signaling and allowing prostate tumor cell death.

SUPPLEMENTARY MATERIALS AND METHODS

Transfection Methods:

Cells were transiently transfected using Lipofectamine 2000 reagent (Invitrogen, Grand Island, NY) according to the manufacturer's instructions. For RNA inhibition experiments, 2.5µl of the appropriate siRNA were used. After removal of the construct-lipid complexes, cells were treated with the appropriate medium and ligand conditions and allowed to incubate at 37C/5% CO2 for 48h before being collected, lysed and analyzed. Experiments were performed in triplicate.

qPCR:

Total cellular RNA was prepared utilizing Trizol (Invitrogen, Carlsbad, CA) based on the manufacturer's protocol. cDNA was synthesized from 1 mg RNA using the iScript cDNA Synthesis Kits (BioRad) as per the manufacturer's protocol. Real-time PCR was run using TaqMan Gene Expression Master Mix (Applied Biosystems, Grand Island, NY) according to manufacturer's recommendations. B-Actin was used as the endogenous expression standard. Data were collected on an Applied Biosystems 7500 Fast machine and analyzed using the relative standard curve method.

Subcellular fractionation

Cells were lysed for 15m, room temperature in 500-900uL of cytoplasmic lysis buffer A (10mM HEPES pH 7.9, 10mM KCl, 0.1mM EDTA, 0.4% IGEPAL) with standard protease and phosphatase inhibitors. The resulting suspension was centrifuged at 16000g for 5m at 4C. Supernatants were transferred to a clean 1.5ml tube and stored at -20C until further use. Pellets were washed thrice with 200-500uL 1X phosphate-buffered saline (Gibco, Thermo Scientific, Walton, MA) (5m, 16000g, 4C), reconstituted in ~150-300uL of 1X SDS Sample Buffer, heated at 90C until it had completely dissolved, cooled to room temperature and stored at -20C until further use.

Immunoprecipitation

Cells were lysed in cytoplasmic lysis buffer as described above and the supernatant and pellet separated accordingly. Protein concentrations were determined using the BCA assay (Thermo Scientific, Walton, MA) according to the manufacturer's instructions. Only supernatants were used for immunoprecipitation reactions. 400ug of protein lysate were used per reaction. The antibody:protein ratio used was 1:200. Immunoprecipitation reactions were carried out overnight at 4C on a rotisserie shaker. Protein G Plus beads (Santa Cruz BioTech, Dallas, TX) were added to each reaction and used as per the manufacturer's specifications. Samples were analysed on 8% polyacrylamide gels by SDS-PAGE.

Western Blotting:

Whole cell extracts were prepared by washing the cells twice in PBS and lysing cells in 100-300 µl cell lysis buffer containing sodium dodecyl sulphate. Proteins were quantitated using a BCA assay (Pierce, Rockford IL) and fractionated using 29:1 acrylamide-bis SDS-PAGE. Electrophoresis was performed at 150 V for 120 min using mini-vertical electrophoresis cells (Mini-PROTEAN 3 Electrophoresis Cell, Bio-Rad, Hercules, CA). The gels were electroblotted for 2 h at 200 mA using a Mini Trans-Blot Electrophoretic Transfer Cell (Bio-Rad) onto 0.2-µm polyvinylidene difluoride membrane (Osmonics, Westborough, MA). The blots were stained with primary antibodies at a dilution of 1:250 or 1:500. The staining was detected by enhanced chemiluminescence (Pierce) after incubation with a peroxidase-labeled secondary antibody (Donkey anti-mouse IgG, Chemicon, Temecula, CA, Goat anti-rabbit IgG, Fc specific, Jackson Immunoresearch, West Grove, PA).

3-[4,5-Dimethylthiazol-2yl]-2,5-diphenyl-tetrazolium bromide (MTT) assay

Cells were cultured in 24-well plates and treated as indicated. Following treatment, each well was incubated with 25 µl of 5 mg/ml 3-[4,5-dimethylthiazol-2yl]-2,5-diphenyl-tetrazolium bromide (MTT; Sigma–Aldrich, St. Louis, MO) for 1 h in a 5% CO2 incubator at 37 °C, which converted the reactants to formazan in actively dividing cells. Proliferation rates were estimated by colorimetric assay reading formazan intensity in a plate reader at 562 nm.

Immunohistochemistry:

Mouse tumors were fixed in 10% buffered formalin (Medical Industries, Richmond IL) for 30 mins at RT, after which the pellet was immersed in 600 µl liquefied agar at 50-60°C. The agar containing the tumor was paraffin-embedded and processed based on established protocols. The paraffin-embedded cell block was then sectioned, and sections were heated to 60°C, and rehydrated in xylene and graded alcohols. Antigen retrieval was performed with 0.1M citrate buffer at pH 6.0 for 20 minutes in a 95% water bath. Slides were allowed to cool for another 20 minutes, followed by sequential rinsing in PBS and 50 mM Tris HCl, pH 7.6, 150 mM NaCl, Tween 20 (0.1%) (TBS-T). Endogenous peroxidase activity was quenched by incubation in TBS-T containing 3% hydrogen peroxide. Each incubation step was carried out at room temperature and was followed by three sequential washes (5 minutes each) in TBS-T. Sections were incubated in primary antibody diluted in TBS-T containing 1% ovalbumin and 1 mg/ml sodium azide (12 hours), followed by incubations with biotinylated secondary antibody for 15 minutes, peroxidase-labeled streptavidin for 15 minutes (LSAB-2 Dako Corp, Carpenteria, CA) and diaminobenzidine and hydrogen peroxide chromogen substrate (Dako Corp. Carpentaria CA) along with DAB enhancer (Signet) for 10 minutes. Slides were counter-stained with hematoxylin and mounted. Negative controls were incubated with the same amount of antihuman polyclonal rabbit IgG in place of primary antibody. We used rabbit anti-ErbB3 CS-12708 (Cell Signalling Technology, Beverly, MA), rabbit polyclonal anti-ErbB3 (C-17; 1:100 dilution) and anti-AR (sc-7305, 1:100 dilution) antibodies from Santa Cruz Biotechnology (Dallas, TX), Ki67 was from DAKO, while the TUNEL kit was from Millipore (Burlington, MA). For negative controls, we used a Universal Rabbit IgG control (DAKO) in place of the primary antibody. Only the epithelial cells were scored. The extent of staining was scored 0 to 2, where 0 represented no staining, 1 represents low (<20% staining), þ1 represent s intermediate (30%–50%), þ1.5 (50%–70%) and þ2 represent high staining (>80%).

Immunofluorescence:

C4-2 or 22Rv1 cells were seeded at 10,000 cells per coverslip and were incubated for 24hrs in FBS medium in a 37^{0}C CO2 incubator. Cells were treated with vehicle or drug for 72h, rinsed with PBST (Phosphate Buffered Saline with 0.05% Tween-20) and fixed with ice-cold methanol for 10 min on ice. They were washed three times with PBST and then blocked with 5% BSA for 1h at room temperature. Primary antibody was diluted 1:100 in 1% BSA and applied to the cells and incubated at 4°C overnight in a humidity chamber. Cells were washed three times with PBST and the Rhodamine conjugated anti-rabbit secondary antibody (Life Technologies, Carlsbad, CA) was diluted 1:500 in PBST and incubated for 1 hr at room temperature in the dark. After washing thrice with cold PBST, coverslips were inverted and mounted onto uncharged glass slides with antifade mounting medium plus DAPI (Life Technologies, Carlsbad, CA).

Analysis of apoptosis using flow cytometry

Cells were grown under desired conditions in 60 mm dishes at 1×10^{6} cells/dish. Flow cytometry was conducted on FACSCalibur (Becton Dickinson Immunocytometry Systems, San Jose, CA, USA). Cells were illuminated with 200 mW of 488 nm light produced by an argon-ion laser and 635 nm light produced by a red-diode laser. Fluorescence was read through a 630/22 nm band-pass filter (for propidium iodide) or a 661/16 nm band-pass filter (for Annexin V-Alexa Fluor 647). Data were collected on 20000 cells as determined by forward and right-angle light scatter and stored as frequency histograms; data used for apoptosis were further analyzed using FlowJo version 7 (TreeStar, FlowJo LLC, Ashland, OR).

SUPPLEMENTARY REFERENCES:

1. Whang YE, Armstrong AJ, Rathmell WK, Godley PA, Kim WY, Pruthi RS, et al. A phase II study of lapatinib, a dual EGFR and HER-2 tyrosine kinase inhibitor, in patients with castration-resistant prostate cancer. Urol Oncol 2013;31(1):82-6.

2. Boysen G, Barbieri CE, Prandi D, Blattner M, Chae SS, Dahija A, et al. SPOP mutation leads to genomic instability in prostate cancer. Elife 2015;4.

3. Selvaraj N, Budka JA, Ferris MW, Jerde TJ, Hollenhorst PC. Prostate cancer ETS rearrangements switch a cell migration gene expression program from RAS/ERK to PI3K/AKT regulation. Mol Cancer 2014;13:61.

4. An J, Wang C, Deng Y, Yu L, Huang H. Destruction of full-length androgen receptor by wild-type SPOP, but not prostate-cancer-associated mutants. Cell Rep 2014;6(4):657-69.

5. An J, Ren S, Murphy SJ, Dalangood S, Chang C, Pang X, et al. Truncated ERG Oncoproteins from TMPRSS2-ERG Fusions Are Resistant to SPOP-Mediated Proteasome Degradation. Mol Cell 2015;59(6):904-16.

6. Tomlins SA, Laxman B, Dhanasekaran SM, Helgeson BE, Cao X, Morris DS, et al. Distinct classes of chromosomal rearrangements create oncogenic ETS gene fusions in prostate cancer. Nature 2007;448(7153):595-9.
